# Influence of intermixing at the Ta/CoFeB interface on spin Hall angle in Ta/CoFeB/MgO heterostructures

**DOI:** 10.1038/s41598-017-00994-z

**Published:** 2017-04-20

**Authors:** Monika Cecot, Łukasz Karwacki, Witold Skowroński, Jarosław Kanak, Jerzy Wrona, Antoni Żywczak, Lide Yao, Sebastiaan van Dijken, Józef Barnaś, Tomasz Stobiecki

**Affiliations:** 1 AGH University of Science and Technology, Department of Electronics, Al. Mickiewicza 30, 30-059 Kraków, Poland; 2 Faculty of Physics, Adam Mickiewicz University, ul. Umultowska 85, 61-614 Poznań, Poland; 3 Singulus Technologies AG, Hanauer Landstrasse 103, Kahl am Main, 63796 Germany; 4 AGH University of Science and Technology, Academic Center of Materials and Nanotechnology, Al. Mickiewicza 30, 30-059 Kraków, Poland; 5 NanoSpin, Department of Applied Physics, Aalto University School of Science, P.O. Box 15100, FI-00076 Aalto, Finland; 6 Institute of Molecular Physics, Polish Academy of Sciences, ul. Smoluchowskiego 17, 60-179 Poznań, Poland

## Abstract

When a current is passed through a non-magnetic metal with strong spin-orbit coupling, an orthogonal spin current is generated. This spin current can be used to switch the magnetization of an adjacent ferromagnetic layer or drive its magnetization into continuous precession. The interface, which is not necessarily sharp, and the crystallographic structure of the nonmagnetic metal can both affect the strength of current-induced spin-orbit torques. Here, we investigate the effects of interface intermixing and film microstructure on spin-orbit torques in perpendicularly magnetized Ta/Co_40_Fe_40_B_20_/MgO trilayers with different Ta layer thickness (5 nm, 10 nm, 15 nm), greater than the spin diffusion length. Effective spin-orbit torques are determined from harmonic Hall voltage measurements performed at temperatures ranging from 20 K to 300 K. We account for the temperature dependence of damping-like and field-like torques by including an additional contribution from the Ta/CoFeB interface in the spin diffusion model. Using this approach, the temperature variations of the spin Hall angle in the Ta underlayer and at the Ta/CoFeB interface are determined separately. Our results indicate an almost temperature-independent spin Hall angle of ${{\boldsymbol{\theta }}}_{{\boldsymbol{SH}}}^{{\boldsymbol{N}}}\approx -{\bf{0.2}}$ in Ta and a strongly temperature-dependent ${{\boldsymbol{\theta }}}_{{\boldsymbol{SH}}}^{{\boldsymbol{N}}}$ for the intermixed Ta/CoFeB interface.

## Introduction

It is well known that spin current induced *via* the spin Hall effect (SHE) in a heavy metallic layer may exert a torque on the magnetic moment of an adjacent ferromagnetic layer^1^. This torque in turn can induce magnetization dynamics, which may be observed by various experimental techniques, namely spin-torque ferromagnetic resonance^2^, spin-orbit-torque-induced magnetization switching^3,4^, domain-wall motion^5,6^ or harmonic Hall voltage measurements^7^. Recent reports indicate that signals detected in the ferromagnetic layer do not refer directly to the spin Hall angle, considered as an *intrinsic* property of the non-magnetic layer and defined as the ratio of the induced spin current density to the charge current density in this layer^2^. Consequently, additional torques due to spin-orbit interaction at the interface between the non-magnetic and ferromagnetic layer are invoked. This interfacial spin-orbit interaction leads to a nonequilibrium spin polarization of electrons at the interface, which in turn gives rise to a spin-orbit torque (SOT). Currently, there is a great interest, both experimental^8^ and theoretical^9^, in interface SOTs. However, it is rather difficult to distinguish experimentally between SOTs originating at the interface and torques that are generated by SHE in the non-magnetic layer. Theoretical calculations of SOTs usually assume a sharp interface^10,11^. This approximation is justified in some cases, but it is generally invalid for amorphous structures. For example, in CoFeB/Ta and CoFeB/W^12^, X-ray and neutron reflectometry^13^ indicate strong intermixing between the two layers, resulting in a relatively wide interface region. When considering the interface contribution to the total SOT, spin transport across the interfaces and possible mechanisms of spin relaxation need to be taken into account. Accordingly, the effect can be captured by an efficiency parameter or, equivalently, an effective spin Hall angle. Since the SOT consists of two components – damping-like and field-like torques – two spin Hall torque efficiencies have been proposed^14^. Investigations of interface atomic ordering and electrical conductivity effects on the spin-orbit torque arising from SHE are crucial for the design of novel spintronic devices utilizing SOTs. High resistance phases of heavy metals are desirable as the spin Hall angle is enhanced. For example, tungsten above a certain thickness shows a phase transition from the high resistivity *β*-W phase to the low resistivity *α*-W phase, which results in a decrease of the spin Hall angle^12,15,16^. A detailed assessment of structural effects on SOTs is complicated by a relatively broad distribution of experimental data from different laboratories and the use of different definitions. For instance, reported values of the spin Hall angle in systems with Ta span between −0.03 and −0.15 (at room temperature)^17–21^. Moreover, one should make a distinction between heterostructures with in-plane and perpendicular magnetic anisotropy (PMA) of the ferromagnetic layer. In the case of structures with PMA (considered in this paper), the harmonic Hall voltage method^22–24^ seems to be the most appropriate because the damping-like and field-like torques are determined based on independent measurements. In our Ta/CoFeB bilayers, no transition between *α*-Ta and *β*-Ta phase is observed, the growth of a given phase is determined exclusively by sputtering conditions. The CoFeB layer exhibits PMA and the Ta buffer layer is thicker than the spin diffusion length, which falls within the range 1.2–2.5 nm^18,25–27^. Since available experimental data on the damping-like torque, converted to effective spin Hall angle, fluctuate between −0.03^17^ and −0.11^20^, we investigate effects caused by the interface and crystallographic structure, which may be responsible for the scattering of reported data. We provide microstructural data and electrical conductivity measurements and show that both physical parameters depend on the Ta underlayer thickness, beyond the range already investigated^3,28–32^. The thinnest Ta layer is amorphous, whereas thicker Ta layers comprise the tetragonal *β* phase. In all cases, interface effects originate from the mixing of Ta and CoFeB causing a relatively thick *interface layer*. Patterned structures of micrometer-dimensions are investigated with harmonic Hall voltage measurements in a wide range of temperatures in order to clearly designate the field-like and damping-like torques. Because of substantial interlayer mixing, we propose to model transport properties by considering the interface as a distinct layer with its own spin diffusion length and spin Hall angle.

## Results

### Structure

**Figure 1:**
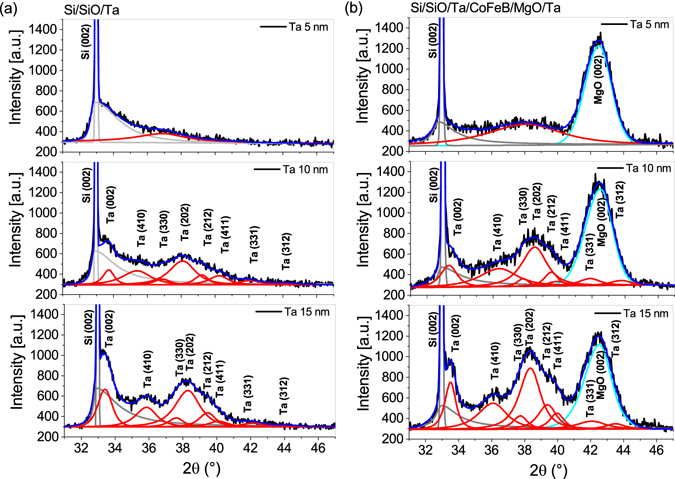
*θ* − 2*θ* profiles for single Ta layers (**a**), and for full multilayer stacks Ta(*d*_Ta_)/Co_40_Fe_40_B_20_(1)/MgO(5)/Ta(3) (**b**), where d_*Ta*_ = 5, 10, 15 nm. Black lines depict the experimental data, blue lines are fits, red lines represent the distribution of Ta peaks for different orientations, cyan lines are a fit for MgO, and gray lines show the substrate peaks.

**Figure 2:**
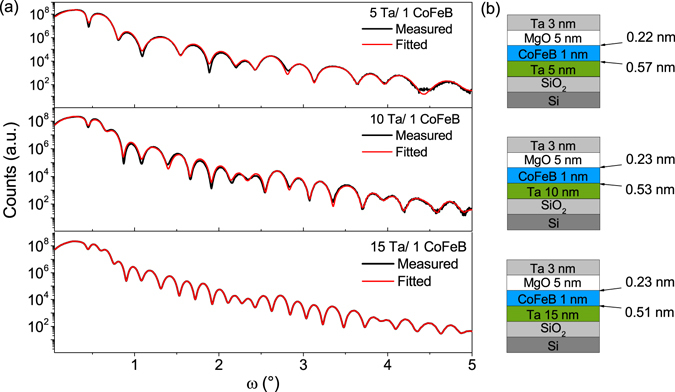
The best fits to X-ray reflectivity data for full structures: Ta(d_Ta_)/CoFeB(1)/MgO(5)/Ta(3), where d_Ta_ = 5, 10, 15 nm (**a**), and interfacial RMS roughness for Ta/CoFeB and CoFeB/MgO interfaces (**b**), determined with an accuracy of ±0.02 nm.

Two sets of samples were prepared. The first set consisted of Ta(*d*_Ta_)/Co_40_Fe_40_B_20_(*t*_*FM*_)/MgO(5)/Ta(3) multilayer stacks, with d_*Ta*_ = 5, 10, 15 and *t*_*FM*_ = 0.8–1.5, while structures in the second set comprised single Ta layers with a thickness of d_*Ta*_ (all thicknesses in nm). Structural analysis of the Ta layer was performed on single Ta layers with *d*_*Ta*_ = 5, 10, 15 nm (Fig. 1(a)) and on annealed Ta(*d*_*Ta*_)/Co_40_Fe_40_B_20_(1)/MgO(5)/Ta(3) multilayer stacks, with identical Ta thickness (Fig. 1(b)). Comparisons between the *θ* − 2*θ* X-ray Diffraction (XRD) profiles of Ta in the full stacks and in single layers do not reveal major structural differences. The *θ* − 2*θ* profiles of 5 nm thick Ta layers show a very broad low-intensity peak, indicating an amorphous-like disordered structure. On the other hand, the profiles of thicker Ta layers (10 nm and 15 nm) contain peaks that originate from a polycrystalline tetragonal *β* phase^33^. The *θ* − 2*θ* XRD measurements on our samples do not indicate the presence of the *α*-Ta phase. For all samples, the MgO layers have a highly (001)-oriented texture, while the thin CoFeB layers remain amorphous after annealing. Subsequently, using the X-ray Reflectivity (XRR) method^34^ we analysed Ta/CoFeB and CoFeB/MgO interfaces; corresponding profiles are presented in Fig. 2(a). The thickness of the CoFeB/MgO interface is about 0.23 nm, while that of the Ta/CoFeB interface is in the range from 0.51 nm to 0.57 nm, see Fig. 2(b). The RMS surface roughness from Atomic Force Microscope (AFM), measured on the surface of single Ta layers, is comparable to the thickness of the CoFeB/MgO interface from XRR. The RMS roughness is as follows: 0.23 nm, 0.26 nm and 0.29 nm for 5 nm, 10 nm and 15 nm of Ta, respectively. As expected, the smoothest surface is found in the amorphous 5 nm Ta sample^35^, while the polycrystalline Ta layers (10 and 15 nm) are increasingly rough. A significant difference between the widths of the Ta/CoFeB and CoFeB/MgO interfaces can be explained by the mechanisms described below. The small thickness of the CoFeB/MgO interface mainly stems from surface roughness, which indicates poor interdiffusion between the CoFeB and MgO layers. In turn, the large thickness of the Ta/CoFeB interface may have an origin in a large negative interfacial enthalpy, which is a driving force for interdiffusion. It is worth noting that for Fe in Ta the interfacial enthalpy is −54 kJ/(mole of atoms) and for Co in Ta it is −86 kJ/(mole of atoms)^36^. The above conclusions are confirmed by the interface change which is observed after annealing by means of XRR analysis. For the annealed samples Ta/CoFeB interface roughness increases by 30 percent with respect to as-deposited layers while the CoFeB/MgO interface remains unaltered. In addition, the XRR measurement results implicate a tendency for the interface thickness to diminish with increasing Ta layer thickness. These phenomena can be explained by easier interdiffusion when both CoFeB and Ta (5 nm) are amorphous than between the amorphous CoFeB and polycrystalline Ta (10 nm and 15 nm) layers. The described tendency is in accordance with the decrease in thickness of the magnetic dead layer, see next paragraph.

**Figure 3:**
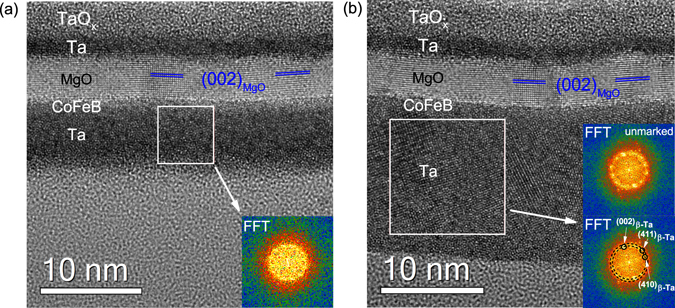
HRTEM images of Ta(*d*_*Ta*_)/CoFeB(1)/MgO(5)/Ta(3) samples. (**a**) *d*_*Ta*_ = 5 nm; (**b**) *d*_*Ta*_ = 15 nm. The (002) planes in the MgO layers are marked with blue lines. The insets in (**a**,**b**) show fast Fourier transform (FFT) patterns from designated areas. The results indicate that the 5 nm Ta layer is amorphous and the 15 nm Ta layer is polycrystalline.

**Figure 4:**
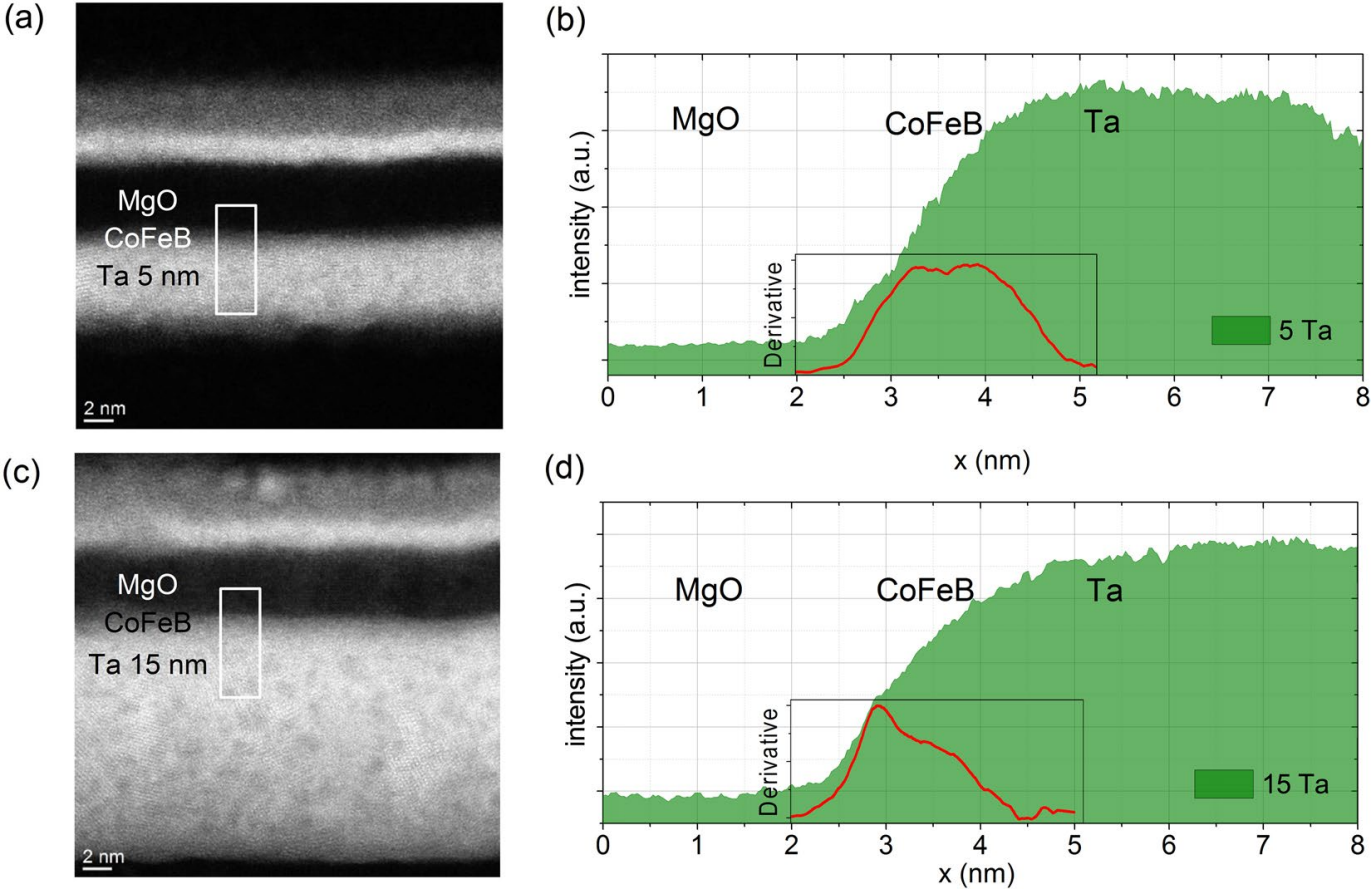
(**a**,**c**) Scanning Transmission Electron Microscopy (STEM) Z-contrast images and (**b**,**d**) Z-contrast profiles from the indicated areas for samples with (**a**,**b**) 5 nm of Ta and (**c**,**d**) 15 nm of Ta. The insets show derivatives of the Z-contrast profiles.

High-resolution Transmission Electron Microscopy (TEM) images of samples with 5 nm Ta and 15 nm of Ta are shown in Fig. 3. The layer thicknesses of each multilayer stack correspond closely to the intended growth parameters. In both samples, the MgO layer exhibits a polycrystalline structure with the (002)-planes oriented parallel to the interfaces, while the CoFeB layer is amorphous. The main difference between the two samples is the crystalline structure of the Ta underlayer. The 5 nm thick Ta layer is amorphous^35^ (Fig. 3(a)), whereas the 15 nm Ta layer is polycrystalline (Fig. 3(b)). The crystallographic orientations in the 15 nm thick Ta layer are in agreement with the *θ* − 2*θ* XRD measurements. The interface between Ta and CoFeB is mixed in both samples. TEM measurements reveal a gradual change in Z-contrast, as illustrated by the line profiles in Fig. 4(b,d). The more gradual increase in Z-contrast in Fig. 4(d) suggests that atomic interdifussion changes slightly when the Ta layer thickness is increased from 5 nm to 15 nm. This effect is most likely caused by the crystal structure of the Ta layers, which evolves from amorphous to polycrystalline when the film becomes thicker. Variations in intermixing and the crystal structure can both affect electronic transport across the Ta/CoFeB interface. In order to apprehend the difference in Z-contrast, we propose the following scenario. For the sample with a 5 nm Ta layer, atomic diffusion takes place between two amorphous layers and thus the intermixed zone is more or less homogeneous. Accordingly, the derivative of the Z-contrast contour is constant in the CoFeB area (inset in Fig. 4(b)). In the case of 15 nm of Ta, however, atomic diffusion takes place between amorphous CoFeB and polycrystalline Ta layers, and intermixing occurs mainly along the Ta grain boundaries. Therefore, the shape of the Z-contrast contour close to Ta is rounded and saturates more slowly (Fig. 4(d)).

### Temperature dependence of electrical and magnetic properties

**Figure 5:**
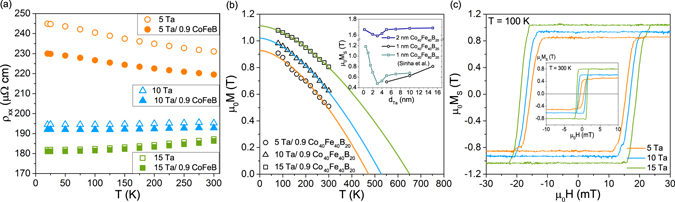
(**a**) Temperature dependence of the longitudinal resistivity for multilayer stacks and single Ta layers. (**b**) Spontaneous magnetization M as a function of temperature; solid lines represent calculations using Bloch’s law; inset: comparison of saturation magnetization M_*S*_ vs. Ta underlayer thickness, for 1 nm and 2 nm of Co_40_Fe_40_B_20_ (our results) and data for 1 nm Co_20_Fe_60_B_20_ in ref.^40^. (**c**) Magnetic hysteresis loops obtained at 100 K and at room temperature (inset).

The longitudinal resistivity *ρ*_*xx*_ of the full multilayer stacks and single Ta layers was measured using a 4-probe method. The room-temperature resistivities of single Ta layers are as follows: *ρ*_5*Ta*_ = 235 *μ*Ω cm, *ρ*_10*Ta*_ = 195 *μ*Ω cm, *ρ*_15*Ta*_ = 185 *μ*Ω cm. The resistivity of Ta can be used as a probe of structural order. High resistivity of more than 200 *μ*Ω cm confirms the amorphous structure of 5 nm Ta. In turn, resistivities of the order of 190 *μ*Ω cm evidence the presence of the *β*-Ta phase in 10 and 15 nm Ta layers^37,38^. The obtained resistivities of *β*-Ta are similar to those reported in refs^18^,^39^. The resistivity of amorphous CoFeB, *ρ*_CoFeB_ ≈ 165 *μ*Ω cm, is derived from the parallel resistors model. The temperature dependence of longitudinal resistivity (*ρ*_*xx*_) for the full stacks and for single Ta layers is presented in Fig. 5(a). Interestingly, the resistivity of the full multilayer stacks changes very little with temperature – the highest resistivity change is 4% for the sample with the thinnest Ta layer. In this case, a negative temperature coefficient of resistivity is noticed, which is characteristic of the amorphous phase. For *d*_*Ta*_ = 5 nm, resistivity of the single layer is noticeably larger than that of the corresponding stack layer. For *d*_*Ta*_ = 10 nm the difference is rather insignificant. All this supports the conjecture that the 10 nm and 15 nm layers of Ta are in the *β* phase, while the 5 nm layer of Ta is amorphous. We examined the magnetization of the CoFeB layer as a function of temperature for samples with different Ta thickness (Fig. 5(b)). The temperature dependence of spontaneous magnetization is described by Bloch’s law: *M* = *M*_0_(1 − (*T*/*T*_*c*_)^3/2^), where *M*_0_ is a spontaneous magnetization at T = 0 K and *T*_*C*_ is the Curie temperature. Strong changes of the CoFeB layer saturation magnetization with Ta thickness provide additional evidence of the amorphous phase and intermixed interface of thin Ta layers. Our saturation magnetization results of 2 nm CoFeB and reports in literature show a drop in saturation magnetization for *d*_*Ta*_ ≈ 3 nm at room temperature (see the inset to Fig. 5(b))^19,40^. This thickness dependence indicates that thin Ta amorphous layers readily mix with amorphous CoFeB, causing a reduction of saturation magnetization. Magnetic hysteresis loops (Fig. 5(c)) confirm perpendicular magnetization in the annealed samples. However, the magnetic hysteresis loop for the sample with 5 nm of Ta shows weaker perpendicular magnetic anisotropy than other samples. This is a result of much smaller interface anisotropy contribution to effective anisotropy^41^ for the sample with 5 nm of amorphous Ta due to a thick magnetic dead layer (MDL). The fact that strong mixing at the Ta/CoFeB interface can result in an MDL has already been reported^40,42,43^. Actually, MDL for the sample with 5 nm of Ta is the widest and reaches 0.55 nm, while for 10 nm of Ta and 15 nm MDL is 0.46 nm and 0.39 nm, respectively. An increased thickness of MDL reflects the interdiffusion at the Ta/CoFeB interface.

### Anomalous Hall effect

**Figure 6:**
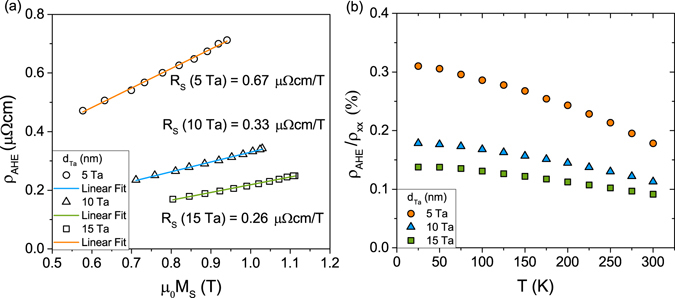
(**a**) Anomalous Hall resistivity of Ta/CoFeB/MgO as a function of saturation magnetization. The slope determines the corresponding anomalous Hall coefficient *R*_*S*_. (**b**) Temperature dependence of the ratio *ρ*_*AHE*_/*ρ*_*xx*_.

Anomalous Hall voltage measurements were performed for an external magnetic field applied perpendicularly to the sample plane and temperatures from 20 K to 300 K. The anomalous Hall effect (AHE) is described quantitatively by the anomalous Hall coefficient *R*_*s*_, which can be calculated using *ρ*_*AHE*_ = *R*_*S*_ · *μ*_0_*M*_*S*_, where *ρ*_*AHE*_ is the anomalous Hall resistivity. Figure 6(a) presents the anomalous Hall resistivity as a function of saturation magnetization. The variation of *M*_*S*_ is derived from temperature-dependent data. The slopes of these curves, which are almost constant, correspond to the anomalous Hall coefficients. This agrees with theoretical predictions^44,45^ showing a proportionality between *R*_*S*_ and *ρ*_*xx*_ (the resistivity of our samples varies less than 4% between 20 K and 300 K (Fig. 5(a))). The larger *R*_*S*_ value for the sample with 5 nm Ta in comparison to other samples points to a substantial influence of the Ta/CoFeB interface. The ratio of the Hall resistivity *ρ*_*AHE*_ to the longitudinal resistivity *ρ*_*xx*_ is shown in Fig. 6(b). The results are comparable to those reported in refs^46,47^. The planar Hall resistance was measured in an external magnetic field applied in the film plane with rotation of the field direction. In contrast to W/CoFeB heterostructures^12,48,49^, the planar Hall effect (PHE) for Ta underlayers is much smaller than the corresponding AHE contribution^19,50^. The ratio of planar Hall resistance, *R*_*PHE*_, and the anomalous Hall resistance, *R*_*AHE*_, is of the order of $\zeta ={R}_{PHE}/{R}_{AHE}\approx \mathrm{0.4 \% }$, which is within the margin of error, and therefore PHE can be omitted. This substantially simplifies the formula for effective torque fields, as described below.

### Spin torque efficiencies

**Figure 7:**
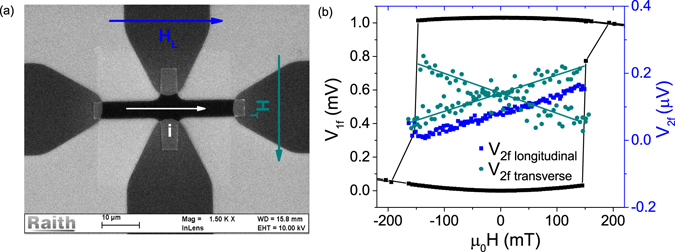
(**a**) SEM image of the Hall bar with illustrated directions of current flow and external magnetic field: longitudinal H_*L*_ and transverse H_*T*_ to the current. (**b**) First harmonic (1f) and second harmonic (2f) Hall voltage signals (longitudinal and transverse in the latter case) measured at 150 K.

**Figure 8:**
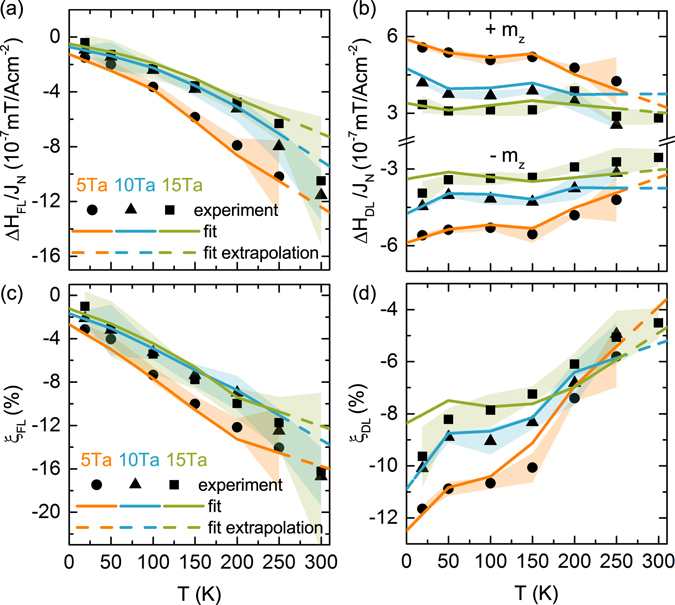
Field-like *H*_*FL*_ (**a**) and damping-like *H*_*DL*_ (**b**) components of the spin-orbit torque induced effective magnetic field. Damping-like components are shown for two indicated orientations of the CoFeB layer magnetization. Spin-torque efficiencies corresponding to the field-like (**c**) and damping-like (**d**) components of the effective magnetic field. Shaded areas denote experimental error bars. The symbols correspond to experimental data while the solid lines represent the numerical fits. Shaded areas denote experimental error bars.

To evaluate the spin torque exerted on the CoFeB layer we determined the spin torque efficiencies. Therefore, the Hall voltage measurements were performed for an external magnetic field applied in the sample plane in two directions: longitudinally and transversely to the current flowing through the Hall bar. Details of the measurement technique are described e.g. in refs^12,22,23^. A lock-in technique was used to measure the first and second harmonics of the Hall voltage. A low frequency (385 Hz) alternating charge current was passed through the Hall bar, while the external magnetic field was applied along and across the Hall bar, as indicated in Fig. 7(a). Measurements were performed on 10 *μ*m wide and 40 *μ*m long Hall bars. In order to determine the current density, the precise widths of the patterned strips were measured by scanning electron microscopy (SEM). From the harmonic Hall voltage measurements, the spin-orbit-induced-effective-fields (related to the spin torques) were obtained as a function of temperature for all three studied samples (for different values of *d*_*Ta*_). From measurements with a longitudinal external magnetic field, *H*_*L*_, we derived the effective field Δ*H*_DL_. Analogously, from measurements with a transverse external field, *H*_*T*_, we obtained the effective field Δ*H*_FL_. Taking into account that $\zeta ={R}_{PHE}/{R}_{AHE}$ is negligibly small, these effective fields are determined by the voltage harmonics according to the formulas

1 $${\rm{\Delta }}{H}_{{\rm{DL}}({\rm{FL}})}=-2\frac{\partial {V}_{{\rm{2f}}}}{\partial {H}_{{\rm{L}}({\rm{T}})}}/\frac{{\partial }^{2}{V}_{{\rm{1f}}}}{\partial {H}_{{\rm{L}}({\rm{T}})}^{2}},$$

where *V*_1*f*,2*f*_ are the first and second harmonic Hall signals measured for *H*_*L*_ and *H*_*T*_. Exemplary voltage signals measured at 150 K are presented in Fig. 7(b). The temperature variation of the effective fields is shown in Fig. 8. Below 150 K, the longitudinal effective field, referred to as the damping-like (DL) field, is approximately constant, while at higher temperatures absolute values slightly decrease in all three samples (see Fig. 8(b)). The transverse effective field, referred to as field-like (FL), steadily decreases with increasing temperature. One can also note that from room temperature down to 150 K, Δ*H*_FL_ dominates. Both fields, Δ*H*_DL_ and Δ*H*_FL_, decrease with increasing Ta layer thickness, contrary to the results reported by Kim *et al*.^19^. However, it should be noted that our results cover a different range of Ta thickness. For thin Ta layers the spin diffusion length has a decisive influence, while for thicker layers studied here the crystallographic structure plays a major role. Taking into account the current density in Ta and magnetic moment of CoFeB, the longitudinal (damping-like) and transverse (field-like) torque efficiences *ξ*_FL(DL)_ are obtained from the formula ${\xi }_{FL(DL)}=(2|e|/\hslash )({\mu }_{0}{M}_{s}{d}_{F}/{J}_{N}){\rm{\Delta }}{H}_{FL(DL)}$. The temperature variation of the evaluated spin torque efficiencies are presented in Fig. 8. The physical meaning of these quantities will be discussed in the next section.

## Discussion

To model spin transport and spin torques we consider interface as a distinct layer with its own properties such as spin diffusion length and spin Hall angle. A similar approach has already been used in the case of Pt/Py structures^11,14^. One of the key issues is the determination of an effective spin Hall angle of the structure, which generally can be a certain function of the spin Hall angle ${\theta }_{SH}^{N}$ of the Ta layer, referred to as a non-magnetic (N) layer, and of the spin Hall angle ${\theta }_{SH}^{I}$ of the interface (I) layer, i.e. ${{\rm{\Theta }}}_{SH}={{\rm{\Theta }}}_{SH}({\theta }_{SH}^{N},{\theta }_{SH}^{I})$. In general, the anomalous Hall effect can also play a role in the conversion of charge current to spin current in N/F bilayer systems. In our case, however, this effect is small, as shown above. A simple drift-diffusion equation for spin current in an N/I/F (Non-magnetic/Interface/Ferromagnetic) structure contains a diffusion term resulting from spin accumulation at the interfaces and a drift term due to SHE (see Theoretical method in Method section). In order to fit this model to experimental data we need to make some assumptions. First of all, we assume a constant spin diffusion length, *λ*_*N*_, in the non-magnetic layer (excluding the interface). In literature this parameter ranges from ~1 nm to more than 3 nm^21,27^. In general, this parameter can also vary with temperature, however we neglect this variation due to possible compensation by changes in resistivity *ρ*_*N*_. Moreover, both real, *G*_*r*_, and imaginary, *G*_*i*_, parts of spin-mixing conductance are fixed by fitting to the data for a range of spin diffusion lengths and spin Hall angles. This fitting shows that, approximately, *G*_*r*_(*T*) ~ *const* and *G*_*i*_(*T*) ~ *T*.

This is consistent with the mixing counductance estimated for bulk Ta by *ab initio* methods^51,52^, where, however, a crystalline phase was assumed and the strong spin-orbit coupling was not taken into account. The model has been then fitted to the experimental data for the available range of temperatures. Furthermore, the so-called spin memory loss (SML) parameter, defined as ${\rm{SML}}=[1-\exp (-{d}_{I}/{\lambda }_{I})]\cdot \mathrm{100 \% }$, has been introduced. In numerical calculations we assumed *d*_*I*_/*λ*_*I*_ = 0.05, which corresponds to ${\rm{SML}}\approx \mathrm{4.9 \% }$. This parameter has been assumed constant with respect to temperature. Such an assumption, however, may not hold at higher temperatures.

Figure 8 show the best fit of the model to the experimental data on the field-like and damping-like components of the effective magnetic field and to the corresponding spin torque efficiencies. The absolute value of the field-like component of the spin-torque efficiency increases with increasing temperature, while the damping-like component decreases. This behaviour of the field-like component can be explained by a dominant contribution of the imaginary part of the spin-mixing conductance. In the case of the damping-like component, the temperature dependence of *g*_*i*_ cancels out and the dominating contribution comes from the temperature dependence of the effective spin Hall angle.

**Figure 9:**
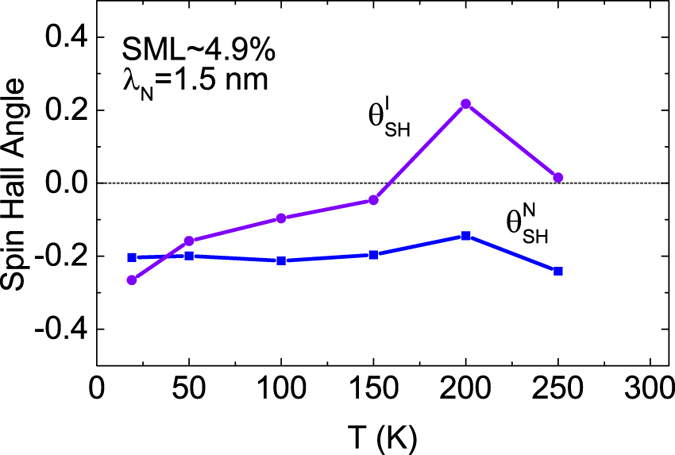
Spin Hall angle of the interface and non-magnetic metal Ta layers obtained from fits to experimental data with indicated parameters of spin memory loss (SML) and *λ*_*N*_.

Figure 9 shows the temperature dependence of the spin Hall angle in the non-magnetic and interface layers, and the results indicate a strong interfacial effect. As the spin Hall angle in the non-magnetic layer is approximately constant with respect to temperature, the interfacial spin Hall angle changes its sign for temperatures between 150–250 K. This sign reversal indicates that the interfacial spin Hall angle should be treated as an effective spin current conversion coefficient, which is influenced by both non-magnetic and ferromagnetic layers, and whose behaviour at higher temperatures may differ from the behaviour of non-magnetic metals. Additional processes may play a role in this temperature range, resulting in higher spin memory loss. Opposite signs of the spin Hall angles in the non-magnetic and interfacial layers have occurred also in the analysis of spin-pumping-induced ISHE in a Bi/Py bilayer system, where scattering on impurities has been considered as a possible explanation of this behaviour^53^. The second interesting feature of the interface spin Hall angle is its magnitude, which in the vicinity of 200 K is remarkably larger (though it has an opposite sign) than that in the Ta layer. This, however, is reasonable, as the charge current density in the interface layer is smaller than that in the Ta layer. Apart from this, an additional extrinsic mechanism of SHE in the interface layer can occur due to intermixing (side-jump and/or skew-scattering on magnetic impurities). Generally, signs (and also magnitudes) of different contributions may be different as well. This, in turn, may lead to sing reversal of the Hall effect in the interface layer. It should be also taken into consideration that for temperature of 250 K the measured effective fields had the greatest uncertainty due to narrow switching characteristic. We also note that the interfacial spin Hall angle in a Pt/Py structure has been estimated to be 25 times larger than the spin Hall angle in a single Pt layer^11^, whereas the interfacial contribution in a Bi/Py bilayer has been estimated to be ca. 4 times larger than the contribution from a single Pt layer^53^.

In conclusion, we examined the spin Hall effect and spin torques in Ta/CoFeB/MgO heterostructures. The crystallographic structure of a non-magnetic metal layer and properties of the interface with a ferromagnetic layer were investigated. We demonstrated that strong intermixing at the interface leads to a magnetically dead layer, which is especially thick for structures with 5 nm of an amorphous Ta layer. To account for the experimentally determined damping-like and field-like effective fields, we applied a drift-diffusion model, assuming the interfacial region as a distinct layer. By fitting the theoretical model to experimental data we have determined the spin Hall angle in the Ta layer and in the Ta/CoFeB interface layer. The interface spin Hall angle was shown to change its sign with increasing temperature. Moreover, at temperatures around 200 K its magnitude is larger than the spin Hall angle in the tantalum layer.

## Methods

### Sample preparations and micro-fabrication

All samples were deposited using magnetron sputtering in a Singulus Timaris PVD Cluster Tool System on thermally oxidized 4-inch Si(001) wafers. The Ta and CoFeB layers were magnetron sputtered under argon pressure of 2.7 × 10^−3^ mbar. For CoFeB, a linear dynamic deposition (LDD) wedge technology was used to achieve a smooth gradation of film thickness. Samples with a different *t*_*FM*_ layer were used to study the formation of a magnetic dead layer. However, for the SHE measurements, all structures had a constant CoFeB thickness, *t*_*FM*_ = 0.91 nm. For this thickness, the as-deposited samples exhibited an uniaxial in-plane anisotropy, which turned into perpendicular anisotropy after 20 minutes of post-deposition annealing at 330 °C. Using e-beam lithography and ion-beam etching methods, the samples were patterned into 10 *μ*m wide and 40 *μ*m long Hall bars with 100 × 100 *μ*m^2^ contact pads. Additional structures were prepared for 4-point resistance measurements. During the microfabrication process, the covering Ta layer was etched so that only a thin naturally oxidized Ta layer was left as a protective layer.

### Measurement method

Continuous layers were used for structural and magnetic characterization. *θ* − 2*θ* X-ray Diffraction was employed to study the crystallograhic structure. Interface roughness was examined by X-ray Reflectivity and the surface morphology of the Ta layers was measured using Atomic Force Microscopy. High-resolution structural characterization of multilayer samples with 5 nm and 15 nm of Ta was carried out using a JEOL 2200FS Transmission Electron Microscope with double Cs correctors. Both HRTEM and STEM with high-angle annular dark-field (HAADF) contrast (Z-contrast) were used. The magnetic properties of the FM layer were probed using a LakeShore 7407 vibrating sample magnetometer (VSM) with an LN2 cryostat. The magnetic dead layer thickness was obtained from the x-axis interception value of the linear fit to M/A (the magnetic moment per unit area) vs. *t*_*FM*_. The change of magnetization was measured in a magnetic field (±50 mT) at temperatures ranging from 80 K to 300 K. Anomalous Hall resistance used for obtaining *ρ*_*AHE*_ was calculated as a ratio of anomalous Hall voltage to current flowing through magnetic parts. Anomalous Hall voltage and spin-orbit effective fields were measured by means of Keithley 2636 A source-meter and Stanford SR830 DSP Lock-in Amplifier.

### Theoretical method

The drift-diffusion equation written down for the non-magnetic (N) and interface (I) layers takes the following form refs^14,19,54,55^:

2 $$\begin{array}{rcl}{{\bf{j}}}_{s}^{N}(z) & = & -\frac{1}{2e{\rho }_{N}}\frac{\partial {{\boldsymbol{\mu }}}_{s}^{N}(z)}{\partial z}-{\theta }_{SH}^{N}{J}_{N}\hat{{\boldsymbol{y}}},\\ {{\bf{j}}}_{s}^{I}(z) & = & -\frac{1}{2e{\rho }_{I}}\frac{\partial {{\boldsymbol{\mu }}}_{s}^{I}(z)}{\partial z}-{\theta }_{SH}^{I}{J}_{I}\hat{{\boldsymbol{y}}},\end{array}$$

where *ρ*_*N*_ and *ρ*_*I*_ denote resistivity of the N and I layers, respectively, *e* is the electron charge (*e* < 0), ***μ***_*s*_ is the spin accumulation, while *J*_*N*_ and *J*_*I*_ denote the charge current density in the N and I layers (along the x axis). In the following we assume ${\theta }_{SH}^{I}=\alpha {\theta }_{SH}^{N}$, with the proportionality coefficient *α* considered as a fitting parameter. Boundary conditions necessary for derivation of the spin accumulation ***μ***_*s*_ (and thus also spin current) take the following form:

3 $$\begin{array}{l}{{\bf{j}}}_{s}^{N}(z={d}_{I})={{\bf{j}}}_{s}^{I}(z={d}_{I}),\\ {{\bf{j}}}_{s}^{I}(z=\mathrm{0)}={{\bf{j}}}_{s}^{F|I},\\ {{\bf{j}}}_{s}^{N}(z={d}_{I}+{d}_{N})=0,\\ {{\boldsymbol{\mu }}}_{s}^{I}(z={d}_{I})={{\boldsymbol{\mu }}}_{s}^{N}(z={d}_{N}),\end{array}$$

where *d*_*I*,*N*_ is the thickness of the I and N layers, respectively, and *z* = 0 corresponds to the I/F interface. Furthermore, ${{\bf{j}}}_{s}^{F|I}$ is the spin current at the F/I interface, taken on the interface layer side. This spin current obeys the boundary condition^51^:

4 $$e{{\bf{j}}}_{s}^{F|I}={G}_{r}\hat{{\bf{m}}}\times \hat{{\bf{m}}}\times {{\boldsymbol{\mu }}}_{s}^{I}(0)+{G}_{i}\hat{{\bf{m}}}\times {{\boldsymbol{\mu }}}_{s}^{I}(0),$$

where $\hat{m}$ is a unit vector along the magnetic moment of the F layer, ${G}_{r}\equiv {\rm{Re}}[{G}_{mix}]$, ${G}_{i}\equiv {\rm{Im}}[{G}_{mix}]$, and *G*_*mix*_ is the so-called spin-mixing conductance. The above equation is appropriate when the spin polarization of SHE-induced spin current is normal to $\hat{m}$. Otherwise the spin current component parallel to $\hat{{\bf{m}}}$ may flow into the magnet and can induce the anomalous Hall effect.

The spin current ${{\bf{j}}}_{s}^{F|I}$ creates a torque in the ferromagnetic layer, which can be expressed in terms of an effective field Δ**H** as

5 $$\frac{\partial \hat{{\bf{m}}}}{\partial t}={\boldsymbol{\tau }}=\gamma \hat{{\bf{m}}}\times {\rm{\Delta }}{\bf{H}},$$

where *γ* is the gyromagnetic ratio and Δ**H** is related to the spin current ${{\bf{j}}}_{s}^{F|I}$ via the formula

6 $${\rm{\Delta }}{\bf{H}}=\frac{\hslash }{2e}\frac{1}{{\mu }_{0}{M}_{s}{d}_{F}}\hat{{\bf{m}}}\times {{\bf{j}}}_{s}^{F|I}.$$

The damping-like, ${\rm{\Delta }}{\bf{H}}\cdot \hat{{\bf{x}}}\equiv {\rm{\Delta }}{H}_{DL}$, and field-like, ${\rm{\Delta }}{\bf{H}}\cdot \hat{{\bf{y}}}\equiv {\rm{\Delta }}{H}_{FL}$ components of this effective field are

7 $$\begin{array}{rcl}{\rm{\Delta }}{H}_{DL} & = & \frac{\hslash }{2e}\frac{{J}_{N}}{{\mu }_{0}{M}_{s}{d}_{F}}{\theta }_{SH}^{N}\\  &  & \times \frac{\tanh (\frac{{d}_{N}}{2{\lambda }_{N}})\mathrm{csch}(\frac{{d}_{I}}{{\lambda }_{I}})+\alpha (\frac{{\rho }_{N}}{{\rho }_{I}})[\tanh (\frac{{d}_{I}}{2{\lambda }_{I}})\coth (\frac{{d}_{N}}{{\lambda }_{N}})-\frac{{\rho }_{I}{\lambda }_{I}}{{\rho }_{N}{\lambda }_{N}}]}{\coth (\frac{{d}_{N}}{{\lambda }_{N}})\coth (\frac{{d}_{I}}{{\lambda }_{I}})+\frac{{\rho }_{I}{\lambda }_{I}}{{\rho }_{N}{\lambda }_{N}}}\\  &  & \times \frac{{g}_{r}(1+{g}_{r})+{g}_{i}^{2}}{{(1+{g}_{r})}^{2}+{g}_{i}^{2}}(-{m}_{z}),\\ {\rm{\Delta }}{H}_{FL} & = & -\frac{\hslash }{2e}\frac{{J}_{N}}{{\mu }_{0}{M}_{s}{d}_{F}}{\theta }_{SH}^{N}\\  &  & \times \frac{\tanh (\frac{{d}_{N}}{2{\lambda }_{N}})\mathrm{csch}(\frac{{d}_{I}}{{\lambda }_{I}})+\alpha (\frac{{\rho }_{N}}{{\rho }_{I}})[\tanh (\frac{{d}_{I}}{2{\lambda }_{I}})\coth (\frac{{d}_{N}}{{\lambda }_{N}})-\frac{{\rho }_{I}{\lambda }_{I}}{{\rho }_{N}{\lambda }_{N}}]}{\coth (\frac{{d}_{N}}{{\lambda }_{N}})\coth (\frac{{d}_{I}}{{\lambda }_{I}})+\frac{{\rho }_{I}{\lambda }_{I}}{{\rho }_{N}{\lambda }_{N}}}\\  &  & \times \frac{{g}_{i}}{{(1+{g}_{r})}^{2}+{g}_{i}^{2}},\end{array}$$

where *m*_*z*_ = ±1 is the projection of the ferromagnet’s magnetization onto the *z* axis and *g*_*r*,*i*_ defined as:

8 $${g}_{r,i}=2{G}_{r,i}\frac{\coth (\frac{{d}_{N}}{{\lambda }_{N}})\coth (\frac{{d}_{I}}{{\lambda }_{I}})+\frac{{\rho }_{I}{\lambda }_{I}}{{\rho }_{N}{\lambda }_{N}}}{\frac{1}{{\rho }_{I}{\lambda }_{I}}\,\coth (\frac{{d}_{N}}{{\lambda }_{N}})+\frac{1}{{\rho }_{N}{\lambda }_{N}}\,\coth (\frac{{d}_{I}}{{\lambda }_{I}})}.$$

## References

[1] Hoffmann A (2013). Spin Hall effects in metals. IEEE Transactions on Magnetics.

[2] Liu L, Moriyama T, Ralph D, Buhrman R (2011). Spin-torque ferromagnetic resonance induced by the spin Hall effect. Physical Review Letters.

[3] Lo Conte R (2014). Spin-orbit torque-driven magnetization switching and thermal effects studied in Ta/CoFeB/MgO nanowires. Applied Physics Letters.

[4] Hao Q, Xiao G (2015). Giant spin Hall effect and switching induced by spin-transfer torque in a W/Co_40_Fe_40_B_20_/MgO structure with perpendicular magnetic anisotropy. Physical Review Applied.

[5] Durrant CJ (2016). Scanning Kerr microscopy study of current-induced switching in Ta/CoFeB/MgO films with perpendicular magnetic anisotropy. Applied Physics Letters.

[6] Emori S, Bauer U, Ahn S-M, Martinez E, Beach GS (2013). Current-driven dynamics of chiral ferromagnetic domain walls. Nature Materials.

[7] Kim K-W, Lee H-W, Lee K-J, Stiles MD (2013). Chirality from interfacial spin-orbit coupling effects in magnetic bilayers. Physical Review Letters.

[8] Fan, X. *et al*. Quantifying interface and bulk contributions to spin–orbit torque in magnetic bilayers. *Nature Communication* **5**, 3042 (2014). 10.1038/ncomms4042 24401766

[9] Amin VP, Stiles MD (2016). Spin transport at interfaces with spin-orbit coupling: Phenomenology. Physical Review B.

[10] Haney PM, Lee H-W, Lee K-J, Manchon A, Stiles MD (2013). Current induced torques and interfacial spin-orbit coupling: Semiclassical modeling. Physical Review B.

[11] Wang L (2016). Giant room temperature interface spin Hall and inverse spin Hall effects. Physical Review Letters.

[12] Skowroński W (2016). Temperature dependence of spin-orbit torques in W/CoFeB bilayers. Physical Review Letters.

[13] Zhu T (2012). The study of perpendicular magnetic anisotropy in CoFeB sandwiched by MgO and tantalum layers using polarized neutron reflectometry. Applied Physics Letters.

[14] Pai C-F, Ou Y, Vilela-Leão LH, Ralph D, Buhrman R (2015). Dependence of the efficiency of spin Hall torque on the transparency of Pt/ferromagnetic layer interfaces. Physical Review B.

[15] Pai C-F (2012). Spin transfer torque devices utilizing the giant spin Hall effect of tungsten. Applied Physics Letters.

[16] Hao Q, Chen W, Xiao G (2015). Beta (*β*) tungsten thin films: structure, electron transport, and giant spin Hall effect. Applied Physics Letters.

[17] Zhang C (2013). Magnetotransport measurements of current induced effective fields in Ta/CoFeB/MgO. Applied Physics Letters.

[18] Allen G, Manipatruni S, Nikonov DE, Doczy M, Young IA (2015). Experimental demonstration of the coexistence of spin Hall and rashba effects in *β*-tantalum/ferromagnet bilayers. Physical Review B.

[19] Kim J (2014). Anomalous temperature dependence of current-induced torques in CoFeB/MgO heterostructures with Ta-based underlayers. Physical Review B.

[20] Qiu, X. *et al*. Angular and temperature dependence of current induced spin-orbit effective fields in Ta/CoFeB/MgO nanowires. *Scientific Reports* **4**, 4491 (2014). 10.1038/srep04491 PMC3967151 24670317

[21] Liu L (2012). Spin-torque switching with the giant spin Hall effect of tantalum. Science.

[22] Pi UH (2010). Tilting of the spin orientation induced by Rashba effect in ferromagnetic metal layer. Applied Physics Letters.

[23] Hayashi M, Kim J, Yamanouchi M, Ohno H (2014). Quantitative characterization of the spin-orbit torque using harmonic Hall voltage measurements. Physical Review B.

[24] Akyol M (2015). Effect of the oxide layer on current-induced spin-orbit torques in Hf/CoFeB/MgO and Hf/CoFeB/TaOx structures. Applied Physics Letters.

[25] Kwon JH, Deorani P, Yoon J, Hayashi M, Yang H (2015). Influence of tantalum underlayer on magnetization dynamics in Ni_81_Fe_19_ films. Applied Physics Letters.

[26] Behera, N., Chaudhary, S. & Pandya, D. K. Anomalous anti-damping in sputtered *β*-Ta/Py bilayer system. *Scientific Reports* **6**, 19488 (2016). 10.1038/srep19488 PMC4726053 26782952

[27] Morota M (2011). Indication of intrinsic spin Hall effect in 4d and 5d transition metals. Physical Review B.

[28] Jamali M (2016). Planar Hall effect based characterization of spin orbital torques in Ta/CoFeB/MgO structures. Journal of Applied Physics.

[29] Hou Q, Xiao G (2015). Giant spin Hall effect and magnetotransport in a Ta/CoFeB/MgO layered structure: A temperature dependence study. Physical Review B.

[30] Kim J (2013). Layer thickness dependence of the current-induced effective field vector in Ta|CoFeB|MgO. Nature Materials.

[31] Ou Y, Pai C-F, Shi S, Ralph D, Buhrman R (2016). Origin of fieldlike spin-orbit torques in heavy metal/ferromagnet/oxide thin film heterostructures. Physical Review B.

[32] Kim J, Sheng P, Takahashi S, Mitani S, Hayashi M (2016). Spin Hall magnetoresistance in metallic bilayers. Physical Review Letters.

[33] Moseley PT, Seabook CJ (1973). The crystal structure of *β*-tantalum. Acta Cryst..

[34] Bowen, D. K. & Tanner, B. K. X-Ray Metrology in Semiconductor Manufacturing. *CRC Press* 115–129 (2006).

[35] Liu J, Ohkubo T, Mitani S, Hono K, Hayashi M (2015). Correlation between the spin Hall angle and the structural phases of early 5d transition metals. Applied Physics Letters.

[36] De Boer, F. R., Mattens, W., Boom, R., Miedema, A. & Niessen, A. Cohesion in metals. North-Holland, Amsterdam (1988).

[37] Clevenger L, Mutscheller A, Harper J, Cabral C, Barmak K (1992). The relationship between deposition conditions, the beta to alpha phase transformation, and stress relaxation in tantalum thin films. Journal of Applied Physics.

[38] Stella K, Bürstel D, Franzka S, Posth O, Diesing D (2009). Preparation and properties of thin amorphous tantalum films formed by small e-beam evaporators. Journal of Physics D: Applied Physics.

[39] Avci CO (2014). Fieldlike and antidamping spin-orbit torques in as-grown and annealed Ta/CoFeB/MgO layers. Physical Review B.

[40] Sinha J (2013). Enhanced interface perpendicular magnetic anisotropy in Ta/CoFeB/MgO using nitrogen doped ta underlayers. Applied Physics Letters.

[41] Tao BS (2014). Perpendicular magnetic anisotropy in Ta/Co_40_Fe_40_B_20_/MgAl_2_O_4_ structures and perpendicular CoFeB/MgAl_2_O_4_/CoFeB magnetic tunnel junction. Applied Physics Letters.

[42] Chen Y-T, Xie S (2012). Magnetic and electric properties of amorphous Co_40_Fe_40_B_20_ thin films. Journal of Nanomaterials.

[43] Frankowski M (2015). Buffer influence on magnetic dead layer, critical current, and thermal stability in magnetic tunnel junctions with perpendicular magnetic anisotropy. Journal of Applied Physics.

[44] Smit J (1958). The spontaneous Hall effect in ferromagnetics II. Physica.

[45] Berger L (1970). Side-jump mechanism for the Hall effect of ferromagnets. Physical Review B.

[46] Wu S, Zhu T, Yang X, Chen S (2013). The anomalous Hall effect in the perpendicular Ta/CoFeB/MgO thin films. Journal of Applied Physics.

[47] Zhu T, Chen P, Zhang Q, Yu R, Liu B (2014). Giant linear anomalous Hall effect in the perpendicular CoFeB thin films. Applied Physics Letters.

[48] Cho S, Park B-G (2015). Large planar Hall effect in perpendicularly magnetized W/CoFeB/MgO structures. Current Applied Physics.

[49] Cho S (2015). Large spin Hall magnetoresistance and its correlation to the spin-orbit torque in W/CoFeB/MgO structures. Scientific Reports.

[50] Torrejon, J. *et al*. Interface control of the magnetic chirality in CoFeB/MgO heterostructures with heavy-metal underlayers. *Nature Communications* **5**, 4655 (2014). 10.1038/ncomms5655 25130480

[51] Brataas A, Bauer GE, Kelly PJ (2006). Non-collinear magnetoelectronics. Physics Reports.

[52] Zwierzycki M, Tserkovnyak Y, Kelly PJ, Brataas A, Bauer GE (2005). First-principles study of magnetization relaxation enhancement and spin transfer in thin magnetic films. Physical Review B.

[53] Hou D (2012). Interface induced inverse spin Hall effect in bismuth/permalloy bilayer. Applied Physics Letters.

[54] Chen Y-T (2013). Theory of spin Hall magnetoresistance. Physical Review B.

[55] Chen Y-T (2016). Theory of spin Hall magnetoresistance (SMR) and related phenomena. Journal of Physics: Condensed Matter.

