# Publisher Correction: Influence of intermixing at the Ta/CoFeB interface on spin Hall angle in Ta/CoFeB/MgO heterostructures

**DOI:** 10.1038/s41598-018-20353-w

**Published:** 2018-02-06

**Authors:** Monika Cecot, Łukasz Karwacki, Witold Skowroński, Jarosław Kanak, Jerzy Wrona, Antoni Żywczak, Lide Yao, Sebastiaan van Dijken, Józef Barnaś, Tomasz Stobiecki

**Affiliations:** 1 AGH University of Science and Technology, Department of Electronics, Al. Mickiewicza 30, 30-059 Kraków, Poland; 2 Faculty of Physics, Adam Mickiewicz University, ul. Umultowska 85, 61-614 Poznań, Poland; 3 Singulus Technologies AG, Hanauer Landstrasse 103, Kahl am Main, 63796 Germany; 4 AGH University of Science and Technology, Academic Center of Materials and Nanotechnology, Al. Mickiewicza 30, 30-059 Kraków, Poland; 5 NanoSpin, Department of Applied Physics, Aalto University School of Science, P.O. Box 15100, FI-00076 Aalto, Finland; 6 Institute of Molecular Physics, Polish Academy of Sciences, ul. Smoluchowskiego 17, 60-179 Poznań, Poland

Correction to: *Scientific Reports* 10.1038/s41598-017-00994-z, published online 20 April 2017

The original version of this Article contained a typographical error in the spelling of the author Sebastiaan van Dijken, which was incorrectly given as Sebastiaan Dijken. This has now been corrected in the PDF and HTML versions of the Article.

